# 3D chromatin architecture and epigenetic regulation in cancer stem cells

**DOI:** 10.1007/s13238-020-00819-2

**Published:** 2021-01-16

**Authors:** Yuliang Feng, Xingguo Liu, Siim Pauklin

**Affiliations:** 1 Botnar Research Centre, Nuffield Department of Orthopaedics, Rheumatology and Musculoskeletal Sciences Old Road, University of Oxford, Oxford, OX3 7LD UK; 2 Guangzhou Regenerative Medicine and Health Guangdong Laboratory, CAS Key Laboratory of Regenerative Biology, Joint School of Life Sciences, Hefei Institute of Stem Cell and Regenerative Medicine, Guangzhou Institutes of Biomedicine and Health, Chinese Academy of Sciences; Guangzhou Medical University, Guangzhou, 510530 China; 3 Guangdong Provincial Key Laboratory of Stem Cell and Regenerative Medicine, Institute for Stem Cell and Regeneration, Guangzhou Institutes of Biomedicine and Health, Chinese Academy of Sciences, Guangzhou, 510530 China

**Keywords:** chromatin architecture, 3D chromatin topology, epigenetics, tumorigenesis, cancer stem cells, pluripotent stem cells

## Abstract

Dedifferentiation of cell identity to a progenitor-like or stem cell-like state with increased cellular plasticity is frequently observed in cancer formation. During this process, a subpopulation of cells in tumours acquires a stem cell-like state partially resembling to naturally occurring pluripotent stem cells that are temporarily present during early embryogenesis. Such characteristics allow these cancer stem cells (CSCs) to give rise to the whole tumour with its entire cellular heterogeneity and thereby support metastases formation while being resistant to current cancer therapeutics. Cancer development and progression are demarcated by transcriptional dysregulation. In this article, we explore the epigenetic mechanisms shaping gene expression during tumorigenesis and cancer stem cell formation, with an emphasis on 3D chromatin architecture. Comparing the pluripotent stem cell state and epigenetic reprogramming to dedifferentiation in cellular transformation provides intriguing insight to chromatin dynamics. We suggest that the 3D chromatin architecture could be used as a target for re-sensitizing cancer stem cells to therapeutics.

## Introduction

The development of many cancers involves a dedifferentiation of cellular identity with the acquisition of a stem cell-like state in a subpopulation of cancer cells. The arising cancer stem cells (CSCs) are exceptionally important because their developmental plasticity allows them to resist conventional therapies, metastasize and give rise to new tumours. The changes in cell identity are caused by transcriptional dysregulation which is a universal feature of tumorigenesis and impacts all cancer hallmarks (Hanahan and Weinberg, 2011). It is increasingly evident that spatiotemporal changes in 3D chromatin architecture have a central function in governing gene transcription and thereby cancer development and cellular heterogeneity.

This review provides an overview of the roles of 3D chromatin architecture in cancer development and progression with an emphasis on the processes that regulate the phenotypic plasticity of cancer stem cells. We argue that early embryonic development and cancer cell dedifferentiation share similar principles in epigenetic regulation and the dynamic changes in 3D chromatin architecture, while representing the opposite direction of the developmental processes. Therefore, 3D chromatin architecture of embryonic stem cells and early lineage specification provide unique insight to the stem cell-like properties of cancer stem cells and intra-tumoural heterogeneity. To strengthen this argument, we draw parallels also with induced pluripotent stem cell (iPSC) generation via reprogramming of 3D chromatin architecture in differentiated cells. In addition, we compare the general features of 3D chromatin architecture in phenotypic plasticity during normal development as well as tumorigenesis, which plays an important role in metastatic spread, therapeutic resistance and tumour relapse. Lastly, we propose therapeutic strategies targeting the 3D chromatin architecture in the maintenance of CSCs stemness for the intervention of cancer progression.

## The Hierarchy of 3D Chromatin Architecture in Mammalian Cells

Enhancers are key cis-regulatory elements that mediate transcription regulation by converging signals from oncogenic and developmental pathways (Hnisz et al., 2015). The clustering of many enhancers on the same DNA molecule that span tens of kilobases has been termed as super-enhancer (Hnisz et al., 2013, Loven et al., 2013). These large regulatory clusters integrate signals from multiple cell fate pathways, and provide strength and robustness to cell-specific gene transcription (Loven et al., 2013; Hnisz et al., 2015) via tissue-specific transcription factors binding (Spitz and Furlong, 2012). Only approximately 7% of enhancers in human cells have been estimated to control their closest promoters while other enhancers can bypass their closest genes and regulate the target promoters through long-range physical communications and thereby impact cellular plasticity and differentiation propensity. In most cases, enhancers interact with their target gene promoters within the same chromosome. However, increasing evidence suggests the existence of interchromosomal enhancer-promoter interactions (Maass et al., 2019). For example, a recent report identified that specific super-enhancers from different chromosomes come into proximity and regulate the transcription essential for identity of olfactory neurons (Monahan et al., 2019).

Mechanistically, enhancer-promoter contact regulates gene expression via increasing transcriptional bursting fraction (more transcriptional events per time frame) but not bursting size (more RNA molecules per transcriptional event) (Bartman et al., 2016). It should be noted that enhancer-promoter contact may not be necessary for active transcription as seen from a recent report showing decreased enhancer-promoter proximity in *Sonic hedgehog* (*Shh*) *gene* activation (Benabdallah et al., 2019), implying that other mechanisms, such as phase separation may be involved in transcription regulation (Misteli, 2020).

Loop extrusion model proposed that topological structures called insulated neighbourhoods can frame and facilitate enhancer-promoter interactions. In this model, cohesin-containing extrusion complex loads onto the DNA and extrudes the DNA loop until blocked by convergently oriented CTCF molecules. This loop extrusion process depends on the hydrolysis of ATP by cohesin’s ATPase activity (Vian et al., 2018; Davidson et al., 2019; Kim et al., 2019). Inhibition of ATP production by oligomycin dramatically disrupted establishment of RAD21 (a cohesin subunit)-associated chromatin loops. In addition, encyclopedia of DNA elements (ENCODE) consortium generated RAD21-associated chromatin contact maps by ChIA-PET (chromatin interaction analysis by paired-end tag sequencing) in 24 human cell lines and identified that 28% of all RAD21 loops are cell-type specific and overlapped with cell-type specific enhancers marked by H3K27ac (Grubert et al., 2020), suggesting that specific enhancer-promoter contacts may be orchestrated by cell-specific insulated neighbourhoods. In addition, insulated neighbourhoods can further aggregate into topologically associating domains (TADs), whose boundaries are demarcated by CTCF binding (Dowen et al., 2014; Hnisz et al., 2016). TADs are clustered into larger domains called A compartments (expression active) and B compartments (expression inactive) (Lieberman-Aiden et al., 2009). However, degradation of CTCF by auxin system only led to the loss of TADs but compartment structures remained largely unchanged, suggesting that the principles governing compartment organization are independent from TADs (Nora et al., 2017). A recent study found that compartment shift is correlated with suppression of the stemness program and tumor progression, and related to DNA hypomethylation (Johnstone et al., 2020). Whether modulation of DNA methylation state can causally alter the compartment switch should be further examined in the future.

Based on cell population Hi-C data, previous studies have demonstrated that TADs remain largely unchanged during cellular specification (Dixon et al., 2012; Nora et al., 2012). However, by single-cell Hi-C approach, a recent study showed that TADs structures vary substantially at single-cell level (Stevens et al., 2017) and CTCF prevents the interactions of inter-TADs (Szabo et al., 2020). Furthermore, by combining single-cell Hi-C and high-resolution microscopy, the authors revealed that TADs can be subdivided into chromatin nanodomains (CND), which depend on nucleosome-nucleosome interactions but are depleted of CTCF or cohesin (Szabo et al., 2020). In trichostatin A (an inhibitor of histone deacetylases) treated cells, TADs remain unchanged but CND organization is largely disrupted, suggesting CND organization relies on the histone acetylation state (Szabo et al., 2020).

## Dedifferentiation in Cancer and Epigenetic Reprogramming

**Figure 1:**
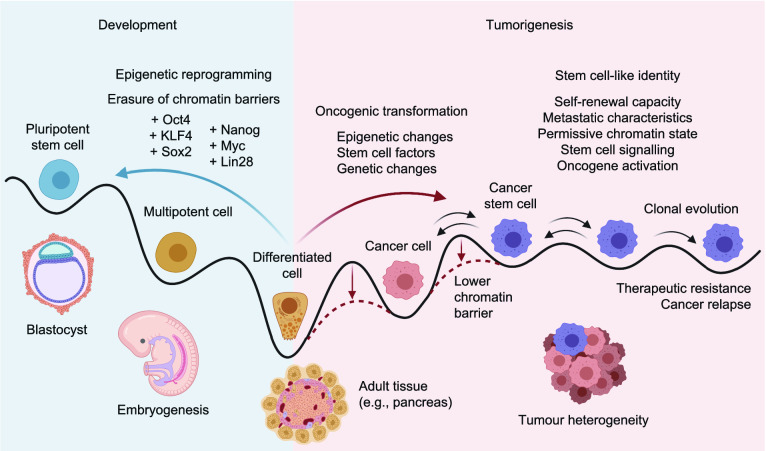
**Formation of cancer stem cells and epigenetic reprogramming**. Schematic depiction of cell state transitions during early development and tumorigenesis indicate dynamic changes according to cell types. Pluripotent stem cells are able to differentiate to all cell types and lead to fully differentiated cells in adult tissues. Epigenetic reprogramming by expression of various stem cell factors (e.g., Oct4, Sox2, KLF4, Myc, Nanog, Lin28) leads to the erasure of the epigenetic barriers and generation of induced pluripotent stem cells. Oncogenic transformation has the opposite direction to normal cell differentiation and the epigenetic changes, expression of stem cell factors and genetic mutations, can facilitate tumorigenesis by lowering the barriers that usually would prevent tumorigenesis and cell state changes. Tumorigenesis involves differentiation of the cell state to a dysregulated stem cell-like identity known as cancer stem cells. These cells are developmentally plastic and can self-renew but also differentiate to other cancer cells

Cancer stem cells or tumour initiating cells exhibit stem cell-like features such as self-renewal and differentiation capacity. CSCs are also resistant to anoikis, a form of programmed cell death when the cells are detached from the surrounding extracellular matrix (ECM). Therefore, the tumorigenic process with dedifferentiation of tissue cells to a stem cell-like or progenitor state seems to be the opposite process to normal development during early embryogenesis and organogenesis (Fig. 1). Dedifferentiation has a similar developmentally reversed direction as the generation of inducible pluripotent stem cells by epigenetic reprogramming. During this reprogramming, exogenous expression of pluripotency factors induces extensive epigenetic remodelling that leads to the activation of an endogenous gene circuitry that maintains the pluripotent state of cells (Papp and Plath, 2013). The resemblance of these two processes—dedifferentiation and epigenetic reprogramming—is highlighted by the effects of tumour suppressors such as p53 and cyclin dependent kinase inhibitors (e.g., p16) in blocking epigenetic reprogramming, while their inactivation increases the epigenetic reprogramming efficiency. p53 is also the most frequently mutated gene across all tumours. For instance, in pancreatic ductal adenocarcinoma it is, together with p16 loss of function, a hallmark mutation.

There is a further link between reprogramming and oncogenic transformation. Transient expression of reprogramming factors *in vivo* in mouse results in tumour development in various tissues consisting of undifferentiated dysplastic cells exhibiting global changes in DNA methylation patterns. This indicates epigenetic regulation associated with reprogramming in the absence of irreversible genetic transformations may drive development of particular types of cancer (Ohnishi et al., 2014). Furthermore, transient expression of reprogramming factors in *Kras* mutant mice is sufficient to induce the robust and persistent activation of ERK signaling in acinar cells and rapid formation of pancreatic ductal adenocarcinoma (Shibata et al., 2018), indicating that reprogramming factors promote oncogenic transformation if the anti-tumorigenic barriers have been removed.

## Regulation of CSC Plasticity by Chromatin Topology

The presence of developmentally plastic cell states with self-renewal capacity has been found in many tumour types (Friedmann-Morvinski et al., 2012; Medema, 2013; Friedmann-Morvinski and Verma, 2014). These stem cell-like cancer cells, CSCs, make up only a small fraction of the whole cancer, but they have the potential to initiate stochastic maturation processes and transitions between differentiated cellular phenotypes. Regardless of the initial differentiation status, cancer cells can re-establish the heterogeneous cell mix when cultured individually (Gupta et al., 2011). As in ESCs, pluripotency factors such as NANOG, OCT4 and SOX2 block cellular differentiation and maturation when expressed in progenitor cells. The unscheduled expression of pluripotency factors can predispose to and drive cancer development. Many genes involved in regulating stem cell functions and stem cell signalling pathways are dysregulated in cancer cells and therefore promote dedifferentiation with the emergence of cancer cells with stem cell-like characteristics. Similar to their function in embryonic stem cells, these genes act at all stages of tumorigenesis by preventing differentiation and eroding barriers against dedifferentiation. For instance, Oct4 maintains the pluripotent state of embryonic stem cells during preimplantation development, but its activation results in dysplastic growths in epithelial tissues with the expansion of progenitor cells and inhibition of differentiation (Hochedlinger et al., 2005) while blocking Oct4 expression leads to apoptosis of CSC populations in human and murine cancer cell lines (Hu et al., 2008). In human lung cancer cells, SOX2 can regulate the transcriptional network of oncogenes and affect lung tumorigenesis (Chen et al., 2012). OCT4 and NANOG enhance malignancy in lung adenocarcinoma by inducing cancer stem cell-like properties and epithelial-mesenchymal transdifferentiation (Chiou et al., 2010), and increased metastasis in breast cancer (Lu et al., 2014). Furthermore, inducible expression of SOX2, OCT4 and KLF4 in melanoma cells leads to partial reprogramming of these cancer cells which start exhibiting increased invasion potential and lung colonization (Knappe et al., 2016). NANOG-positive cells also exhibit enhanced ability of self-renewal, clonogenicity, and initiation of tumors, which are consistent with crucial hallmarks in the definition of CSCs (Shan et al., 2012) and its increased expression in cancer cells is correlated with a worse clinical outcome in hepatocellular carcinoma (HCC) (Shan et al., 2012). It is hypothesized that OCT4, NANOG, KLF4 and SOX2 form extensive feed-forward and feedback loops to organize a stem-cell-like transcriptional enhancer circuitry in ESCs, which could also be mechanistically responsible for inducing stem cell-like characteristics, block expression of differentiation genes, and increase cellular heterogeneity of cancer cells. Although the mechanisms of pluripotency factors in the transcription regulation of CSCs remained largely unexplored, recent studies showed that KLF4 is the key transcription factor mediating the rewiring of H3K27ac-marked enhancer connectome during iPSC reprogramming (Di Giammartino et al., 2019) and SOX2 orchestrates RNAPII-associating chromatin interactions in neural progenitor cells (Bertolini et al., 2019). These studies imply that pluripotency factors may possibly regulate CSC identity through modulating enhancer-promoter interactions.

In addition to pluripotency factors, architectural proteins can also impact cell identity and possibly cancer stem cell formation. The loss of Insulated neighborhoods or TADs boundary has been well documented in carcinogenesis, which arise from structural variation or loss of CTCF binding due to DNA methylation (Hnisz et al., 2018). Addition to CTCF and cohesin, Brother of The Regulator of Imprinted Sites (BORIS, also known as CTCFL) is an emerging architectural protein, which is a paralog of CTCF and typically expressed in testis and ESCs (Loukinov et al., 2002) but aberrantly overexpressed in several cancers (D’Arcy et al., 2008; Bhan et al., 2011; Link et al., 2013; Cheema et al., 2014). BORIS expression is correlated with their risk status, tumor stage, presence of cancer stem cells expressing CD133 marker, and response to targeted therapy (Debruyne et al., 2019). Like CTCF, BORIS regulates gene expression through modulating chromatin looping. In ALK inhibitor sensitive versus resistant neuroblastoma cancer cells, BORIS regulated chromatin loopings specific in resistant cells were associated with the formation of specific super enhancer formation that drive expression of a group of transcription factors that define the resistance phenotype (Debruyne et al., 2019).

Besides the core pluripotency factors and architectural proteins, other transcription factors and components of the epigenetic machinery impact stemness characteristics of cancer cells. Polycomb complex protein Bmi-1 promotes invasion and metastasis of pancreatic CSCs (Wang et al., 2016). The YB1 transcription factor induces the stemness-related gene expression and epithelial-mesenchymal transition in hepatocellular carcinoma (Chao et al., 2017) while YY1 is associated with transcription factor (SOX2, OCT4, BMI1) expression in cancers suggesting a co-regulatory role in cancer stem cells (Kaufhold et al., 2016; Johnson et al., 2019). The ubiquitously expressed transcription factor YY1 has been found to bind on active enhancer and promoter in mESCs. Depletion of YY1 led to reduced contact frequency of enhancer-promoter and decreased gene expression (Weintraub et al., 2017). Localization of YY1 on enhancers and promoters depends on its binding with chromatin-associated RNAs (Sigova et al., 2015), which underlie their potential roles in YY1-mediated enhancer-promoter looping.

MEIS1 is a key transcription factor orchestrating transcriptional programs for the maintenance of CSC plasticity in acute myeloid leukemia (AML). A recent study revealed that MEIS1 is regulated by a frequently interacting region (FIRE) whose intensity of interaction with MEIS1 promoter is linked to heterogenous expression level of *MEIS1* in AML patients (Wang et al., 2020). Deletion of the FIRE in an AML cell line led to transcription repression of *MEIS1* and reduced cell growth.

In addition, in hormone-responsive breast cancers, progestins increase the population of cancer stem cells through the actions of transcription factor C/EBPα. In CD44^+^CD24^−^ breast cancer stem cells, C/EBPα assists progesterone receptor (PR) binding via maintenance of chromatin opening prior to PR binding (Nacht et al., 2019). In these regions, C/EBPα facilitates enhancer-promoter communications for the genes involved in progestin-regulated cell proliferation through interaction with architectural proteins RAD21, YY1 and CTCF. Knockdown of C/EBPα reduced the enrichment of architectural proteins on these regions and disrupted enhancer-promoter interactions (Nacht et al., 2019), highlighting the key role of C/EBPα in the regulation of 3D genome topology in breast cancer stem cells.

Moreover, a recent study identified a lncRNA, Cancer stem cell associated distal enhancer of SOX2 (*CASCADES*) as a novel epigenetic regulator for glioma cancer stem cells (GCSCs) (Shahzad et al., 2020). Hi-C mapping revealed the *CASCADES* intronic enhancer interacted with SOX2 promoter in GCSCs. Knockdown of *CASCADES* in GCSCs led to reduced enrichment of RAD21, YY1 and RNAPII on *CASCADES* intronic enhancer and *SOX2* promoter. As enhancer RNA can bind to RNA-binding protein (RBP) and boost enhancer-promoter looping via RBP dimerization (Cai et al., 2020), whether *CASCADES* functions in a similar mechanism to regulate *SOX2* expression needs to be further examined.

Collectively, the plasticity of CSCs might be acquired and maintained by stem cell factors, epigenetic machineries and stem cell signalling pathways that regulate gene expression via epigenetic processes. These stemness-promoting epigenetic events are likely to involve chromatin architecture at multiple levels: from enhancers/promoters, enhancer-promoter interactions and higher-order genome organisation in the nucleus.

## The Impact of Tumor Microenvironment on 3D Chromatin Architecture in CSCS

CSCs reside in a specialized tumor microenvironment (i.e., niche) which provides essential cues for the self-renewal and propensity of CSCs. Hypoxia is considered to be a major feature of tumour niche which regulate the phenotype of CSCs. Culture in hypoxia can expand the CD133^+^ glioma cancer stem cells through activation of hypoxia inducible factor 1α (HIF1α) (Soeda et al., 2009). Abrogation of HIF1α can eliminate cancer stem cells in hematological malignancies (Wang et al., 2011; Zhang et al., 2012). As HIF1α activate gene transcription central to metabolic rewiring and stemness in CSC, future efforts are required to uncover whether HIF1α induces *de novo* enhancer-promoter interactions in CSCs or just acts on pre-established enhancer-promoter interactions as seen for heat shock factor 1 (HSF1) (Ray et al., 2019).

Perivascular niche is another key component in tumour microenvironment, which comprises endothelial cells and other stromal cells. For example, endothelial cells maintain the phenotype of brain tumour stem cells through secretion of diffusible factors. Accumulating data indicated that microvesicles or exosomes are essential carriers for intercellular communication, such as transfer of RNA. Recent studies demonstrated that chromatin-interacting RNAs are largely involved in enhancer-promoter/promoter-promoter interactions (Li et al., 2017; Cai et al., 2020). In addition, deletion of RNA binding of CTCF dramatically reduced CTCF-mediated chromatin looping (Hansen et al., 2019; Saldana-Meyer et al., 2019), suggesting that chromatin-interacting RNAs are important regulators in 3D genome organization. Hence it would be appealing to interrogate whether perivascular niche-derived exosomal RNA can be transferred into the nucleus of CSCs and regulate the 3D chromatin architecture. A recent study revealed that cohesin-mediated loop extrusion is an ATP-dependent process (Vian et al., 2018; Kim et al., 2019). In addition, mitochondria, the major organelle for ATP production, can be transferred from one cell to another via tunnelling nanotubes (TNTs) or microvesicles (Plotnikov et al., 2015) (e.g., the transfer from leukemic microenvironment to leukemic stem cells (Griessinger et al., 2017)). It is plausible to speculate that the import of mitochondria from niche will reprogram the ATP pool and reorganize 3D chromatin topology in CSCs.

In addition, interstitial ECM provides physical and mechanical cues to drive cancer stemness. For example, collagen I induces EMT through nuclear translocation of β-catenin (Li et al., 2010), a transcription factor coordinating enhancer-promoter looping for MYC (Yochum et al., 2010). Increasing ECM stiffness leads to activation of the LYN kinase, which phosphorylates the EMT transcription factor TWIST1 for nuclear translocation, thus triggering EMT and cancer progression (Fattet et al., 2020). ECM can also transmit the tension force into the nucleus via cytoskeleton, LINC complexes (SUN and KASH domain proteins) in the nuclear membrane, and the nuclear lamina (Osmanagic-Myers et al., 2015), thus reprogramming gene expression. Intriguingly, softer ECM favours the growth of nuclear condensate while stiffer ECM inhibits the growth of nuclear condensate, suggesting that the impact of mechanical forces on nuclear transcription might be mediated by acting on nuclear condensates. As growing evidence suggested that phase separation is a driver for 3D genome organization (Misteli, 2020), how the rigidity of ECM affects the 3D chromatin architecture in CSCs via phase separation deserves future investigation.

## Similarities in Chromatin Architecture in Pluripotent Stem Cells and Cancer Stem Cells?

**Figure 2:**
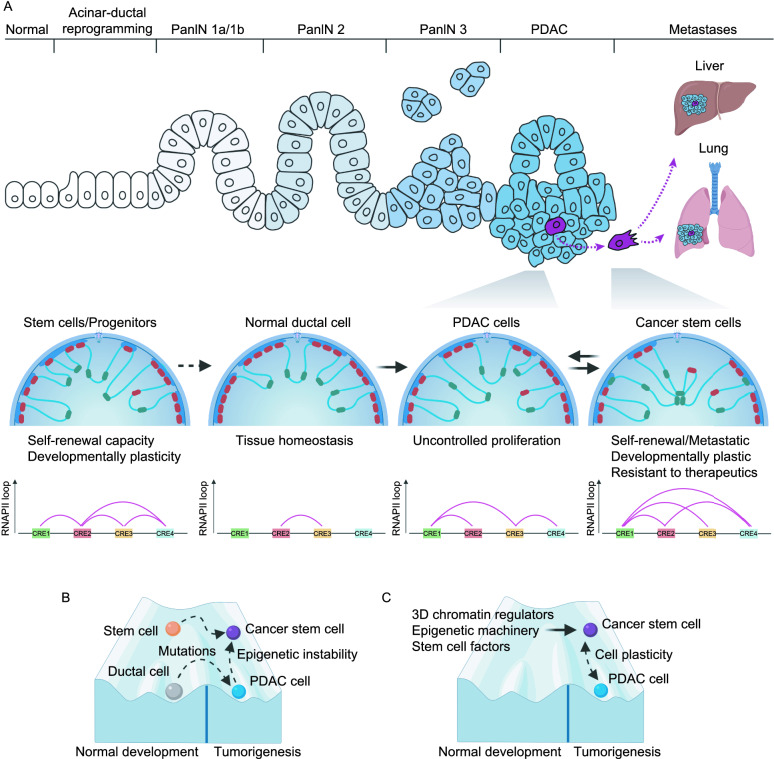
**Targeting 3D chromatin architecture in CSCs**. (A) Rearrangement of the higher-order epigenome during tumorigenesis leads to changes in the cell state from differentiated cell to cancer cells and to cancer stem cells (CSCs). Pancreatic ductal adenocarcinoma (PDAC) formation occurs by gradual transitioning of normal ductal cells to pancreatic intraepithelial neoplasia (PanIN) lesions that ultimately leads to PDAC formation. The PDAC is a heterogenous cell population containing cancer stem cells (CSCs) that can self-renew, are developmental plasticity, highly metastatic and more resistant to conventional therapeutics than other cancer cells. The simplified depiction of the higher-order epigenome inside the nuclei during tumorigenesis indicating rearrangements of active (green) and inactive (red) chromatin regions as well as lamina-associated domains (blue). The genome is organized into topologically associated domains that form gene expression domains with enhancer-promoter crosstalk. Different chromatin regions are postulated to be brought together in different cell states such as CSCs by transcription factors and regulatory proteins that facilitate chromatin accessibility and gene expression regulation. The differences in cell states are postulated to manifest at the higher-order but also lower-order levels of chromatin that bind RNAPII and sequence-specific transcription factors (pink lines show interaction clusters), as depicted schematically by four *cis*-regulatory elements (CREs). (B) The Waddington landscape of normal tissue development and tumorigenesis. In normal tissue formation, stem cells or progenitor cells differentiated to genetically and epigenetically stable cell types, such as ductal cells in pancreatic tissue. Genomic instability due to the accumulation of mutations in driver genes, and epigenomic instability leads to the formation of CSCs and non-CSCs either from normal stem cells or differentiated ductal cells, or through further dedifferentiation of non-CSCs to CSCs. (C) The phenotypic plasticity of CSCs and non-CSCs depicted on the Waddington landscape. The plasticity of CSCs allows the cells to give rise to the whole tumour in its entire cellular heterogeneity while non-CSCs are less plastic. The plasticity is hypothetically regulated by the interplay between 3D epigenome, epigenetic machinery, stem cell factors and structural proteins that regulate gene expression

During cancer development and progression, we hypothesize that the dedifferentiation of cells with expression of some stem cell factors leads to the reorganisation of epigenetic signatures and 3D chromatin architecture in developmentally plastic cancer cells (Fig. 2). The arising cells might share some or dysregulated features seen also in normal ESCs. Interestingly, the chromatin organisation of embryonic stem cells has several unique characteristics: they possess open chromatin characteristics and display chromatin hyperplasticity that distinguishes them from somatic cells.

Nuclei of hESCs have high physical plasticity that stiffens upon differentiation (Pajerowski et al., 2007), a decondensed less compact chromatin (Bartova et al., 2008; Lorzadeh et al., 2016) with an enrichment of active histone modifications (Bartova et al., 2008) and considerably decreased heterochromatin, including peripheral heterochromatin (Underwood et al., 2017). The nuclear lamina in ESCs is largely devoid of Lamin A, less organized and more wrinkled (Pagliara et al., 2014). hESCs have high levels of transcriptional activity and are considerably depleted for heterochromatin-associated histone modifications, particularly the Polycomb-associated H3K27me3, which spreads during differentiation and specification to distinct cell types (Efroni et al., 2008; Zhu et al., 2013). Structural proteins such as heterochromatin protein 1 (HP1), the linker histone H1, histone H3.3 and the core histones display highly dynamic kinetics in their association with chromatin in ESCs, further opening the chromatin structure (Azuara et al., 2006; Meshorer et al., 2006; Schlesinger et al., 2017). Reprogrammed pluripotent stem cells also have increased dynamic association of a heterochromatin protein HP1b compared to differentiated cells (Meshorer et al., 2006; Manukyan and Singh, 2014). Conversely, the open chromatin of ESCs requires the involvement of chromatin repressive complexes in protecting against inappropriate transcription of differentiation factors, and for the ordered repression of pluripotency-associated genes during differentiation. Developmental specification of hESCs is accompanied by progressive chromatin restriction from dynamic remodeling to generalized compaction with decreased transcriptional activity. This is accompanied by the reorganization of the histone variant H2A.Z from a broad distribution in hESCs to more restricted regions in promoters and distal regulatory elements in differentiated cells (Zhu et al., 2013), and formation of large H3K9me3-positive heterochromatic foci (Meshorer et al., 2006).

Deep sequencing of cancers has revealed the occurrence of mutations in epigenetic machinery, including regulators of DNA methylation, histone modification and chromatin organization, across a wide variety of cancer types. Recurrent somatic mutations in genes encoding chromatin-associated proteins (polycomb repressive complexes, HDAC1- and HDAC2-containing complexes) indicate that a deregulated chromatin environment can play a causal role in cancer (You and Jones, 2012; Laugesen and Helin, 2014). Cancers also often have aberrant promoter CpG island hypermethylation and transcriptional silencing of tumor suppressor genes and pro-differentiation factors. In ESCs, these genes are held in a “transcription-ready” state mediated by a “bivalent” promoter chromatin pattern consisting of H3K27me3 (repressive) and H3K4me3 (active) histone marks. Embryonic carcinoma cells have two additional repressive marks, H3K9me2 and H3K9me3, both associated with DNA hypermethylation in adult cancers (Ohm et al., 2007; Schlesinger et al., 2007). In human cancer, promoters with a high H3K27me3:H3K4me3 ratio are particularly susceptible to DNA hypermethylation (Dunican et al., 2020). These results suggest that tumor-specific targeting of *de novo* methylation is pre-programmed by an established epigenetic system that normally has a role in marking embryonic genes for repression. Altogether, transient silencing of regulatory genes in stem or progenitor cells with a bivalent mark may leave these genes vulnerable to aberrant DNA hypermethylation and heritable gene silencing during tumour initiation and progression.

Cellular differentiation leads to the formation of large organized chromatin K9 modifications (LOCKs) comprising repressive H3K9me2 and H3K9me3 modifications, and together with DNA methylation, these regulate cell type-specific repression of developmental loci (Wen et al., 2009). LOCKs are largely absent from ESCs but also cancer cell lines, indicating that this could support the phenotypic plasticity of these cells. In line with this notion, epigenetic reprogramming during iPSC generation erases methylation and chromatin states in part by the OCT4-mediated recruitment of H3K9me2 histone demethylase and chromatin remodelling complexes (Shakya et al., 2015). Interestingly, during epithelial-to-mesenchymal transition (EMT), KDM1A-mediated global loss of H3K9me2 at LOCKs occurs first, and is followed by tumour growth factor-β (TGFβ)-induced EMT (McDonald et al., 2011). The EMT also generates cells with properties of stem cells among cancer cell (Mani et al., 2008) hence suggesting a mechanism between EMT-dependent changes in 3D chromatin architecture and the establishment of stem cell features with cellular plasticity in cancer. The loss of H3K9me2/me3 in chromatin regions in cancer cells overlaps with hypomethylated blocks and the location of increased variability in genes that are important for tumorigenic processes or development in a range of cancer types (Berman et al., 2011; Pujadas and Feinberg, 2012; Timp et al., 2014). Thus, the impairment to stabilize and orchestrate the dynamics of chromatin could give rise to cancer cells with unstable phenotypes and incomplete differentiation, some of which would acquire the capacity of self-renewal by expressing stem cell factors and represent cells known as CSCs.

## Targeting 3D Chromatin Architecture in CSCS

### Targeting stemness factors-mediated super enhancer connectome in CSCs

Cancer cells can establish *de novo* oncogenic super-enhancers that induce proliferation. In turn, disruption of super-enhancers can selectively inhibit tumour oncogenes (Loven et al., 2013). Since super-enhancers of stem cells receive signals from the extracellular milieu and this regulates stem cell/progenitor plasticity and lineage choice (Adam et al., 2015), it is possible that similar microenvironmental effects play important roles also for super-enhancer regulation in cancer cells, thus underlining the importance of the CSC niche. While targeting the tumour microenvironment or CSC niche is considered as a potential cancer therapeutic and alternative strategy could be to directly target the super enhancers and epigenetic machineries in CSCs that control the expression of key oncogenes or stemness regulators, thereby blocking CSC self-renewal capacity and developmental plasticity. This would reduce the metastatic spreading of CSCs and cancer relapse if this strategy would be combined with conventional therapeutic strategies that would aim to target the main hallmarks of cancers.

Several factors known to play a central role in embryonic stem cells and used in epigenetic reprogramming, such as pluripotency factors OCT4, NANOG, SOX2 and Klf4, have been reported to be expressed in subpopulations of cancer cells from different tumour types (Hochedlinger et al., 2005; Feinberg et al., 2006; Lengner et al., 2007; Doi et al., 2009; Fischedick et al., 2014; Lu et al., 2014; Marucci et al., 2014; Ohnishi et al., 2014; Xiong et al., 2018). This indicates an aberrant and partial reactivation of stem cell circuitries in cancer stem cells that usually would regulate the self-renewal and pluripotency of embryonic stem cells during early embryogenesis. Such stem cell factor expression has tumorigenic effects due to effects resembling their function in stem cells such as stem cell maintenance and self-renewal which block differentiation. Pluripotency gene loci form contacts with each other in *cis* and in *trans* in the nucleoplasm and utilize shared cell type-specific transcriptional machineries (de Wit et al., 2013). Pluripotency factors are involved in regulating the genomic architecture of pluripotent stem cells. Super enhancers bound by pluripotency factors OCT4, NANOG, SOX2 and KLF4 colocalize in the pluripotent cell nucleus (Whyte et al., 2013) and establish the prevalent regulatory hubs (Novo et al., 2018). In line with this, recent study uncovered that KLF4 is a key driver for the organization of pluripotency-associated three-dimensional enhancer networks which are disassembled during differentiation (Denholtz et al., 2013; Di Giammartino et al., 2019).

The expression of pluripotency factors such as OCT4, NANOG and SOX2 in CSCs could similarly promote the emergence of stem-like cell states by reprogramming of 3D enhancer—promoter interaction that otherwise would prevent developmental plasticity and maintain differentiated cell states. Targeting the stem cell factors not only would reduce cancer risk, but also antagonize the growth of the primary tumour and metastatic derivatives. Tryptophan derivatives and the AhR signalling pathway regulate the transcription of Oct4 and cancer cell stemness, opening a new therapeutic avenue to target stem-like cancer cells (Cheng et al., 2015). Hence, these mechanisms are intriguing targets for small molecule compounds that would aim to reverse the developmental plasticity of CSCs and trap it to a more restricted cell fate that would re-sensitise these cancer cells to combined chemotherapeutic treatments. This would target the epigenetic instability and the emergence of CSCs during tumour evolution and formation of therapeutic resistance.

### Forced 3D genome repositioning in CSCs

Approximately 36% of A/B compartments switch from one type to another as hESCs differentiate into lineages (Dixon et al., 2015) and many pluripotency loci become re-positioned close to the nuclear lamina as they get repressed (Peric-Hupkes et al., 2010). In the reverse-directional process, the reprogramming of cells into induced pluripotent stem cells re-established the pluripotent genome topology including A/B compartments and enhancer-promoter interactions at pluripotency loci (Beagan et al., 2016; Krijger et al., 2016). For example, downregulation of *OCT4* coincides with the formation of compact chromatin at the lamina during differentiation (Ahmed et al., 2010), indicating the compaction process itself could lead to repression (Eskeland et al., 2010; Therizols et al., 2014; Joshi et al., 2015). The localization of genomic regions to the repressive conditions at the nuclear envelope is promoted by transcriptional repressors (Harr et al., 2015), DNA methylation-binding proteins (Guarda et al., 2009), factors that deposit and recognize repressive histone marks H3K9me2/me3 and H3K27me3 (Towbin et al., 2012; Harr et al., 2015), and components of the nuclear envelope (Harr et al., 2015). In turn, the lamina modulates chromatin states by attracting repressive epigenetic modifiers that maintain a repressive environment at the nuclear periphery (Finlan et al., 2008; Lemaitre and Bickmore, 2015; van Steensel and Belmont, 2017). These epigenetic modifiers balance self-renewal and differentiation, affect epigenetic reprogramming and cancer development (Flavahan et al., 2017) indicating that similar spatiotemporal chromatin compartmentalization mechanisms occur in these counter-directional cellular processes. Hence, epigenetic modifying enzymes repressing differentiation-induced genes by relocating them to the nuclear periphery (KDM1A, EHMT2, HDAC3, N-CoR) could be considered an option for diminishing the plasticity and stemness of CSCs.

Recently, a new approach called CRISPR-genome organization (CRISPR-GO) was developed for 3D genome engineering. Mechanistically, the ABI protein will be fused with dCas9 and recruited to the locus of interest via guide RNA. Simultaneously, the PYL1 protein will be fused with the protein specific to the nuclear compartment (e.g., lamina-associating domains (LADs)). Through heterodimerization of ABI and PYL1 by adding ABA, the guide RNA targeted locus will be relocated into the intended compartment (Wang et al., 2018). This powerful method enables us to examine the functional consequence of forced 3D genome repositioning of the genes encoding stemness factors in CSCs. As chromatin compaction is influenced by phase separation, whether and how the artificial tethering force of CRIPSR-GO can overcome the impact of phase separation deserves future investigation.

## Future Directions

In this article, we discussed the recent findings on chromatin architecture and explored their potential mechanisms in shaping transcriptional dysregulation in CSC formation. A comparison of pluripotent stem cells and the heterogeneous cancer cell populations continues to provide intriguing insight into the regulatory landscapes of phenotypic plasticity and helps uncovering novel mechanisms that provide CSCs their stem cell-like characteristics and resistance to cancer therapeutics.

**Figure 3:**
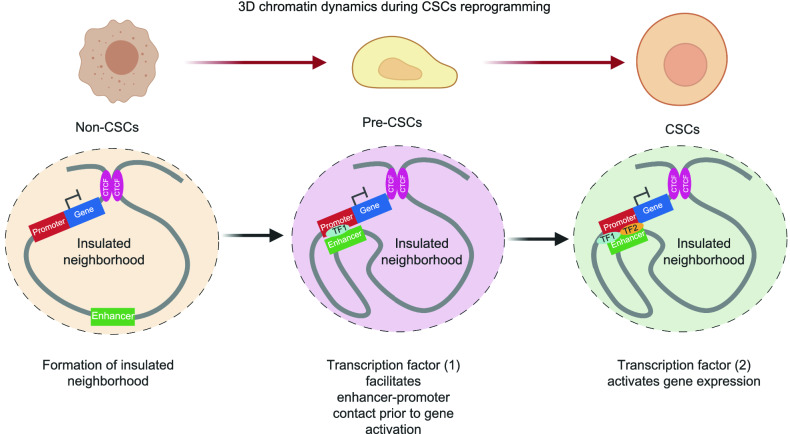
**Hypothesis on the pre-establishment of 3D chromatin architectures during CSCs reprogramming**. As a presumption, a portion of chromatin interactions (e.g., enhancer-promoter contacts) regulating cell fate decision of CSCs may be pre-established in pre-cancerous stem cells (pre-CSCs) via transcription factor (1) binding. These chromatin interaction changes occur prior to the gene transcription. Transcription factor (2) binding on those loci with pre-established enhancer-promoter contacts drives the gene transcription implicated for CSCs reprogramming, leading to the formation of CSCs

Currently, the 3D chromatin dynamics during CSCs dedifferentiation remain elusive. A recent study uncovered that a portion of enhancer-promoter interactions orchestrating human epidermal keratinocyte differentiation were pre-established in the progenitor cells prior to gene expression (Rubin et al., 2017). In addition, glucocorticoid receptors (GR) do not induce *de novo* enhancer-promoter contacts but act on the genes with pre-established enhancer-promoter interactions (D’Ippolito et al., 2018). Moreover, the alteration of gene expression with RAD21-associated loop variation are modest, suggesting that a portion of chromatin topology have not immediate functional impact on gene expression but are poised to alter gene expression in specific developmental and chromatin contexts (Grubert et al., 2020). Hence, future work on the 3D genome mapping in non-CSCs, pre-cancerous stem cells (pre-CSCs) and CSCs can demystify whether enhancer-promoter contacts or insulated neigbourhoods are pre-established in earlier stages for genes that are essential for CSCs dedifferentiation, prior to their transcription activation (Fig. 3).

Recent explosion of single cell-omics techniques enables decoding the unexplored population of CSCs and their niche cells related to tumour initiation and therapeutic resistance. However, the 3D genome mapping for primary CSCs and the niche cells remains challenging due to their limited cell number in isolated tumours. Recently, a new method called HiCAR (high-throughput chromosome conformation capture on Accessible DNA with mRNA-seq co-assay) can simultaneously map the transcriptome, accessible regulatory elements and their interactions in a single assay using only 100,000 cells (Wei et al., 2020), dramatically reducing the input materials as compared to traditional techniques (e.g., HiChIP (Mumbach et al., 2016) /PLAC-seq (Fang et al., 2016) /*in situ* ChIA-PET (Bertolini et al., 2019)). Different from these proximity-ligation based approaches, another method called genome architecture mapping (GAM) can examine multiplex chromatin interactions at single-cell resolution by performing nucleus cryosectioning and sequencing of DNA on nuclear slices (Beagrie et al., 2017). Future efforts (e.g., reducing sectioning thickness) might be able to further increase its resolution.

Collectively, this review article will motivate the study of 3D chromatin architecture in CSCs for a better understanding of their transcription regulation and for manipulating their 3D chromatin topology to improve the therapeutic sensitivity in cancers.

## References

[CR1] Adam RC, Yang H, Rockowitz S, Larsen SB, Nikolova M, Oristian DS, Polak L, Kadaja M, Asare A, Zheng D (2015). Pioneer factors govern super-enhancer dynamics in stem cell plasticity and lineage choice. Nature.

[CR2] Ahmed K, Dehghani H, Rugg-Gunn P, Fussner E, Rossant J, Bazett-Jones DP (2010). Global chromatin architecture reflects pluripotency and lineage commitment in the early mouse embryo. PLoS ONE.

[CR3] Azuara V, Perry P, Sauer S, Spivakov M, Jorgensen HF, John RM, Gouti M, Casanova M, Warnes G, Merkenschlager M (2006). Chromatin signatures of pluripotent cell lines. Nat Cell Biol.

[CR4] Bartman CR, Hsu SC, Hsiung CC, Raj A, Blobel GA (2016). Enhancer regulation of transcriptional bursting parameters revealed by forced chromatin looping. Mol Cell.

[CR5] Bartova E, Krejci J, Harnicarova A, Kozubek S (2008). Differentiation of human embryonic stem cells induces condensation of chromosome territories and formation of heterochromatin protein 1 foci. Differentiation.

[CR6] Bartova E, Galiova G, Krejci J, Harnicarova A, Strasak L, Kozubek S (2008). Epigenome and chromatin structure in human embryonic stem cells undergoing differentiation. Dev Dyn.

[CR7] Beagan JA, Gilgenast TG, Kim J, Plona Z, Norton HK, Hu G, Hsu SC, Shields EJ, Lyu X, Apostolou E (2016). Local genome topology can exhibit an incompletely rewired 3D-folding state during somatic cell reprogramming. Cell Stem Cell.

[CR8] Beagrie RA, Scialdone A, Schueler M, Kraemer DC, Chotalia M, Xie SQ, Barbieri M, de Santiago I, Lavitas LM, Branco MR (2017). Complex multi-enhancer contacts captured by genome architecture mapping. Nature.

[CR9] Benabdallah NS, Williamson I, Illingworth RS, Kane L, Boyle S, Sengupta D, Grimes GR, Therizols P, Bickmore WA (2019). Decreased enhancer-promoter proximity accompanying enhancer activation. Mol Cell.

[CR10] Berman BP, Weisenberger DJ, Aman JF, Hinoue T, Ramjan Z, Liu Y, Noushmehr H, Lange CP, van Dijk CM, Tollenaar RA (2011). Regions of focal DNA hypermethylation and long-range hypomethylation in colorectal cancer coincide with nuclear lamina-associated domains. Nat Genet.

[CR11] Bertolini JA, Favaro R, Zhu Y, Pagin M, Ngan CY, Wong CH, Tjong H, Vermunt MW, Martynoga B, Barone C (2019). Mapping the global chromatin connectivity network for Sox2 function in neural stem cell maintenance. Cell Stem Cell.

[CR12] Bhan S, Negi SS, Shao C, Glazer CA, Chuang A, Gaykalova DA, Sun W, Sidransky D, Ha PK, Califano JA (2011). BORIS binding to the promoters of cancer testis antigens, MAGEA2, MAGEA3, and MAGEA4, is associated with their transcriptional activation in lung cancer. Clin Cancer Res.

[CR13] Cai Z, Cao C, Ji L, Ye R, Wang D, Xia C, Wang S, Du Z, Hu N, Yu X (2020). RIC-seq for global in situ profiling of RNA–RNA spatial interactions. Nature.

[CR14] Chao HM, Huang HX, Chang PH, Tseng KC, Miyajima A, Chern E (2017). Y-box binding protein-1 promotes hepatocellular carcinoma-initiating cell progression and tumorigenesis via Wnt/beta-catenin pathway. Oncotarget.

[CR15] Cheema Z, Hari-Gupta Y, Kita GX, Farrar D, Seddon I, Corr J, Klenova E (2014). Expression of the cancer-testis antigen BORIS correlates with prostate cancer. Prostate.

[CR16] Chen S, Xu Y, Chen Y, Li X, Mou W, Wang L, Liu Y, Reisfeld RA, Xiang R, Lv D (2012). SOX2 gene regulates the transcriptional network of oncogenes and affects tumorigenesis of human lung cancer cells. PLoS ONE.

[CR17] Cheng J, Li W, Kang B, Zhou Y, Song J, Dan S, Yang Y, Zhang X, Li J, Yin S (2015). Tryptophan derivatives regulate the transcription of Oct4 in stem-like cancer cells. Nat Commun.

[CR18] Chiou SH, Wang ML, Chou YT, Chen CJ, Hong CF, Hsieh WJ, Chang HT, Chen YS, Lin TW, Hsu HS (2010). Coexpression of Oct4 and Nanog enhances malignancy in lung adenocarcinoma by inducing cancer stem cell-like properties and epithelial-mesenchymal transdifferentiation. Cancer Res.

[CR19] D’Arcy V, Pore N, Docquier F, Abdullaev ZK, Chernukhin I, Kita GX, Rai S, Smart M, Farrar D, Pack S (2008). BORIS, a paralogue of the transcription factor, CTCF, is aberrantly expressed in breast tumours. Br J Cancer.

[CR20] Davidson IF, Bauer B, Goetz D, Tang W, Wutz G, Peters JM (2019). DNA loop extrusion by human cohesin. Science.

[CR21] de Wit E, Bouwman BA, Zhu Y, Klous P, Splinter E, Verstegen MJ, Krijger PH, Festuccia N, Nora EP, Welling M (2013). The pluripotent genome in three dimensions is shaped around pluripotency factors. Nature.

[CR22] Debruyne DN, Dries R, Sengupta S, Seruggia D, Gao Y, Sharma B, Huang H, Moreau L, McLane M, Day DS (2019). BORIS promotes chromatin regulatory interactions in treatment-resistant cancer cells. Nature.

[CR23] Denholtz M, Bonora G, Chronis C, Splinter E, de Laat W, Ernst J, Pellegrini M, Plath K (2013). Long-range chromatin contacts in embryonic stem cells reveal a role for pluripotency factors and polycomb proteins in genome organization. Cell Stem Cell.

[CR24] Di Giammartino DC, Kloetgen A, Polyzos A, Liu Y, Kim D, Murphy D, Abuhashem A, Cavaliere P, Aronson B, Shah V (2019). KLF4 is involved in the organization and regulation of pluripotency-associated three-dimensional enhancer networks. Nat Cell Biol.

[CR25] D’Ippolito AM, McDowell IC, Barrera A, Hong LK, Leichter SM, Bartelt LC, Vockley CM, Majoros WH, Safi A, Song L (2018). Pre-established chromatin interactions mediate the genomic response to glucocorticoids. Cell Syst.

[CR26] Dixon JR, Selvaraj S, Yue F, Kim A, Li Y, Shen Y, Hu M, Liu JS, Ren B (2012). Topological domains in mammalian genomes identified by analysis of chromatin interactions. Nature.

[CR27] Dixon JR, Jung I, Selvaraj S, Shen Y, Antosiewicz-Bourget JE, Lee AY, Ye Z, Kim A, Rajagopal N, Xie W (2015). Chromatin architecture reorganization during stem cell differentiation. Nature.

[CR28] Doi A, Park IH, Wen B, Murakami P, Aryee MJ, Irizarry R, Herb B, Ladd-Acosta C, Rho J, Loewer S (2009). Differential methylation of tissue- and cancer-specific CpG island shores distinguishes human induced pluripotent stem cells, embryonic stem cells and fibroblasts. Nat Genet.

[CR29] Dowen JM, Fan ZP, Hnisz D, Ren G, Abraham BJ, Zhang LN, Weintraub AS, Schujiers J, Lee TI, Zhao K (2014). Control of cell identity genes occurs in insulated neighborhoods in mammalian chromosomes. Cell.

[CR30] Dunican DS, Mjoseng HK, Duthie L, Flyamer IM, Bickmore WA, Meehan RR (2020). Bivalent promoter hypermethylation in cancer is linked to the H327me3/H3K4me3 ratio in embryonic stem cells. BMC Biol.

[CR31] Efroni S, Duttagupta R, Cheng J, Dehghani H, Hoeppner DJ, Dash C, Bazett-Jones DP, Le Grice S, McKay RD, Buetow KH (2008). Global transcription in pluripotent embryonic stem cells. Cell Stem Cell.

[CR32] Eskeland R, Leeb M, Grimes GR, Kress C, Boyle S, Sproul D, Gilbert N, Fan Y, Skoultchi AI, Wutz A (2010). Ring1B compacts chromatin structure and represses gene expression independent of histone ubiquitination. Mol Cell.

[CR33] Fang R, Yu M, Li G, Chee S, Liu T, Schmitt AD, Ren B (2016). Mapping of long-range chromatin interactions by proximity ligation-assisted ChIP-seq. Cell Res.

[CR34] Fattet L, Jung HY, Matsumoto MW, Aubol BE, Kumar A, Adams JA, Chen AC, Sah RL, Engler AJ, Pasquale EB (2020). Matrix rigidity controls epithelial-mesenchymal plasticity and tumor metastasis via a mechanoresponsive EPHA2/LYN complex. Dev Cell.

[CR35] Feinberg AP, Ohlsson R, Henikoff S (2006). The epigenetic progenitor origin of human cancer. Nat Rev Genet.

[CR36] Finlan LE, Sproul D, Thomson I, Boyle S, Kerr E, Perry P, Ylstra B, Chubb JR, Bickmore WA (2008). Recruitment to the nuclear periphery can alter expression of genes in human cells. PLoS Genet.

[CR37] Fischedick G, Wu G, Adachi K, Arauzo-Bravo MJ, Greber B, Radstaak M, Kohler G, Tapia N, Iacone R, Anastassiadis K (2014). Nanog induces hyperplasia without initiating tumors. Stem Cell Res.

[CR38] Flavahan WA, Gaskell E, Bernstein BE (2017). Epigenetic plasticity and the hallmarks of cancer. Science.

[CR39] Friedmann-Morvinski D, Verma IM (2014). Dedifferentiation and reprogramming: origins of cancer stem cells. EMBO Rep.

[CR40] Friedmann-Morvinski D, Bushong EA, Ke E, Soda Y, Marumoto T, Singer O, Ellisman MH, Verma IM (2012). Dedifferentiation of neurons and astrocytes by oncogenes can induce gliomas in mice. Science.

[CR41] Griessinger E, Moschoi R, Biondani G, Peyron JF (2017). Mitochondrial transfer in the leukemia microenvironment. Trends Cancer.

[CR42] Grubert F, Srivas R, Spacek DV, Kasowski M, Ruiz-Velasco M, Sinnott-Armstrong N, Greenside P, Narasimha A, Liu Q, Geller B (2020). Landscape of cohesin-mediated chromatin loops in the human genome. Nature.

[CR43] Guarda A, Bolognese F, Bonapace IM, Badaracco G (2009). Interaction between the inner nuclear membrane lamin B receptor and the heterochromatic methyl binding protein, MeCP2. Exp Cell Res.

[CR44] Gupta PB, Fillmore CM, Jiang G, Shapira SD, Tao K, Kuperwasser C, Lander ES (2011). Stochastic state transitions give rise to phenotypic equilibrium in populations of cancer cells. Cell.

[CR45] Hanahan D, Weinberg RA (2011). Hallmarks of cancer: the next generation. Cell.

[CR46] Hansen AS, Hsieh TS, Cattoglio C, Pustova I, Saldana-Meyer R, Reinberg D, Darzacq X, Tjian R (2019). Distinct classes of chromatin loops revealed by deletion of an RNA-binding region in CTCF. Mol Cell.

[CR47] Harr JC, Luperchio TR, Wong X, Cohen E, Wheelan SJ, Reddy KL (2015). Directed targeting of chromatin to the nuclear lamina is mediated by chromatin state and A-type lamins. J Cell Biol.

[CR48] Hnisz D, Abraham BJ, Lee TI, Lau A, Saint-Andre V, Sigova AA, Hoke HA, Young RA (2013). Super-enhancers in the control of cell identity and disease. Cell.

[CR49] Hnisz D, Schuijers J, Lin CY, Weintraub AS, Abraham BJ, Lee TI, Bradner JE, Young RA (2015). Convergence of developmental and oncogenic signaling pathways at transcriptional super-enhancers. Mol Cell.

[CR50] Hnisz D, Weintraub AS, Day DS, Valton AL, Bak RO, Li CH, Goldmann J, Lajoie BR, Fan ZP, Sigova AA (2016). Activation of proto-oncogenes by disruption of chromosome neighborhoods. Science.

[CR51] Hnisz D, Schuijers J, Li CH, Young RA (2018). Regulation and dysregulation of chromosome structure in cancer. Annu Rev Cancer Biol.

[CR52] Hochedlinger K, Yamada Y, Beard C, Jaenisch R (2005). Ectopic expression of Oct-4 blocks progenitor-cell differentiation and causes dysplasia in epithelial tissues. Cell.

[CR53] Hu T, Liu S, Breiter DR, Wang F, Tang Y, Sun S (2008). Octamer 4 small interfering RNA results in cancer stem cell-like cell apoptosis. Cancer Res.

[CR54] Johnson TG, Schelch K, Mehta S, Burgess A, Reid G (2019). Why be one protein when you can affect many? The multiple roles of YB-1 in lung cancer and mesothelioma. Front Cell Dev Biol.

[CR55] Johnstone SE, Reyes A, Qi Y, Adriaens C, Hegazi E, Pelka K, Chen JH, Zou LS, Drier Y, Hecht V (2020). Large-scale topological changes restrain malignant progression in colorectal cancer. Cell.

[CR56] Joshi O, Wang SY, Kuznetsova T, Atlasi Y, Peng T, Fabre PJ, Habibi E, Shaik J, Saeed S, Handoko L (2015). Dynamic reorganization of extremely long-range promoter-promoter interactions between two states of pluripotency. Cell Stem Cell.

[CR57] Kaufhold S, Garban H, Bonavida B (2016). Yin Yang 1 is associated with cancer stem cell transcription factors (SOX2, OCT4, BMI1) and clinical implication. J Exp Clin Cancer Res.

[CR58] Kim Y, Shi Z, Zhang H, Finkelstein IJ, Yu H (2019). Human cohesin compacts DNA by loop extrusion. Science.

[CR59] Knappe N, Novak D, Weina K, Bernhardt M, Reith M, Larribere L, Holzel M, Tuting T, Gebhardt C, Umansky V (2016). Directed dedifferentiation using partial reprogramming induces invasive phenotype in melanoma cells. Stem Cells.

[CR60] Krijger PH, Di Stefano B, de Wit E, Limone F, van Oevelen C, de Laat W, Graf T (2016). Cell-of-origin-specific 3D genome structure acquired during somatic cell reprogramming. Cell Stem Cell.

[CR61] Laugesen A, Helin K (2014). Chromatin repressive complexes in stem cells, development, and cancer. Cell Stem Cell.

[CR62] Lemaitre C, Bickmore WA (2015). Chromatin at the nuclear periphery and the regulation of genome functions. Histochem Cell Biol.

[CR63] Lengner CJ, Camargo FD, Hochedlinger K, Welstead GG, Zaidi S, Gokhale S, Scholer HR, Tomilin A, Jaenisch R (2007). Oct4 expression is not required for mouse somatic stem cell self-renewal. Cell Stem Cell.

[CR64] Li A, Zhou T, Guo L, Si J (2010). Collagen type I regulates beta-catenin tyrosine phosphorylation and nuclear translocation to promote migration and proliferation of gastric carcinoma cells. Oncol Rep.

[CR65] Li X, Zhou B, Chen L, Gou LT, Li H, Fu XD (2017). GRID-seq reveals the global RNA-chromatin interactome. Nat Biotechnol.

[CR66] Lieberman-Aiden E, van Berkum NL, Williams L, Imakaev M, Ragoczy T, Telling A, Amit I, Lajoie BR, Sabo PJ, Dorschner MO (2009). Comprehensive mapping of long-range interactions reveals folding principles of the human genome. Science.

[CR67] Link PA, Zhang W, Odunsi K, Karpf AR (2013). BORIS/CTCFL mRNA isoform expression and epigenetic regulation in epithelial ovarian cancer. Cancer Immun.

[CR68] Lorzadeh A, Bilenky M, Hammond C, Knapp D, Li L, Miller PH, Carles A, Heravi-Moussavi A, Gakkhar S, Moksa M (2016). Nucleosome density ChIP-seq identifies distinct chromatin modification signatures associated with MNase accessibility. Cell Rep.

[CR69] Loukinov DI, Pugacheva E, Vatolin S, Pack SD, Moon H, Chernukhin I, Mannan P, Larsson E, Kanduri C, Vostrov AA (2002). BORIS, a novel male germ-line-specific protein associated with epigenetic reprogramming events, shares the same 11-zinc-finger domain with CTCF, the insulator protein involved in reading imprinting marks in the soma. Proc Natl Acad Sci USA.

[CR70] Loven J, Hoke HA, Lin CY, Lau A, Orlando DA, Vakoc CR, Bradner JE, Lee TI, Young RA (2013). Selective inhibition of tumor oncogenes by disruption of super-enhancers. Cell.

[CR71] Lu X, Mazur SJ, Lin T, Appella E, Xu Y (2014). The pluripotency factor nanog promotes breast cancer tumorigenesis and metastasis. Oncogene.

[CR72] Maass PG, Barutcu AR, Rinn JL (2019). Interchromosomal interactions: a genomic love story of kissing chromosomes. J Cell Biol.

[CR73] Mani SA, Guo W, Liao MJ, Eaton EN, Ayyanan A, Zhou AY, Brooks M, Reinhard F, Zhang CC, Shipitsin M (2008). The epithelial-mesenchymal transition generates cells with properties of stem cells. Cell.

[CR74] Manukyan M, Singh PB (2014). Epigenome rejuvenation: HP1beta mobility as a measure of pluripotent and senescent chromatin ground states. Sci Rep.

[CR75] Marucci L, Pedone E, Di Vicino U, Sanuy-Escribano B, Isalan M, Cosma MP (2014). beta-Catenin fluctuates in mouse ESCs and is essential for Nanog-mediated reprogramming of somatic cells to pluripotency. Cell Rep.

[CR76] McDonald OG, Wu H, Timp W, Doi A, Feinberg AP (2011). Genome-scale epigenetic reprogramming during epithelial-to-mesenchymal transition. Nat Struct Mol Biol.

[CR77] Medema JP (2013). Cancer stem cells: the challenges ahead. Nat Cell Biol.

[CR78] Meshorer E, Yellajoshula D, George E, Scambler PJ, Brown DT, Misteli T (2006). Hyperdynamic plasticity of chromatin proteins in pluripotent embryonic stem cells. Dev Cell.

[CR79] Misteli T (2020). The self-organizing genome: principles of genome architecture and function. Cell.

[CR80] Monahan K, Horta A, Lomvardas S (2019). LHX2- and LDB1-mediated trans interactions regulate olfactory receptor choice. Nature.

[CR81] Mumbach MR, Rubin AJ, Flynn RA, Dai C, Khavari PA, Greenleaf WJ, Chang HY (2016). HiChIP: efficient and sensitive analysis of protein-directed genome architecture. Nat Methods.

[CR82] Nacht AS, Ferrari R, Zaurin R, Scabia V, Carbonell-Caballero J, Le Dily F, Quilez J, Leopoldi A, Brisken C, Beato M (2019). C/EBPalpha mediates the growth inhibitory effect of progestins on breast cancer cells. EMBO J.

[CR83] Nora EP, Lajoie BR, Schulz EG, Giorgetti L, Okamoto I, Servant N, Piolot T, van Berkum NL, Meisig J, Sedat J (2012). Spatial partitioning of the regulatory landscape of the X-inactivation centre. Nature.

[CR84] Nora EP, Goloborodko A, Valton AL, Gibcus JH, Uebersohn A, Abdennur N, Dekker J, Mirny LA, Bruneau BG (2017). Targeted degradation of CTCF decouples local insulation of chromosome domains from genomic compartmentalization. Cell.

[CR85] Novo CL, Javierre BM, Cairns J, Segonds-Pichon A, Wingett SW, Freire-Pritchett P, Furlan-Magaril M, Schoenfelder S, Fraser P, Rugg-Gunn PJ (2018). Long-range enhancer interactions are prevalent in mouse embryonic stem cells and are reorganized upon pluripotent state transition. Cell Rep.

[CR86] Ohm JE, McGarvey KM, Yu X, Cheng L, Schuebel KE, Cope L, Mohammad HP, Chen W, Daniel VC, Yu W (2007). A stem cell-like chromatin pattern may predispose tumor suppressor genes to DNA hypermethylation and heritable silencing. Nat Genet.

[CR87] Ohnishi K, Semi K, Yamamoto T, Shimizu M, Tanaka A, Mitsunaga K, Okita K, Osafune K, Arioka Y, Maeda T (2014). Premature termination of reprogramming in vivo leads to cancer development through altered epigenetic regulation. Cell.

[CR88] Osmanagic-Myers S, Dechat T, Foisner R (2015). Lamins at the crossroads of mechanosignaling. Genes Dev.

[CR89] Pagliara S, Franze K, McClain CR, Wylde G, Fisher CL, Franklin RJM, Kabla AJ, Keyser UF, Chalut KJ (2014). Auxetic nuclei in embryonic stem cells exiting pluripotency. Nat Mater.

[CR90] Pajerowski JD, Dahl KN, Zhong FL, Sammak PJ, Discher DE (2007). Physical plasticity of the nucleus in stem cell differentiation. Proc Natl Acad Sci USA.

[CR91] Papp B, Plath K (2013). Epigenetics of reprogramming to induced pluripotency. Cell.

[CR92] Peric-Hupkes D, Meuleman W, Pagie L, Bruggeman SW, Solovei I, Brugman W, Graf S, Flicek P, Kerkhoven RM, van Lohuizen M (2010). Molecular maps of the reorganization of genome-nuclear lamina interactions during differentiation. Mol Cell.

[CR93] Plotnikov EY, Babenko VA, Silachev DN, Zorova LD, Khryapenkova TG, Savchenko ES, Pevzner IB, Zorov DB (2015). Intercellular transfer of mitochondria. Biochemistry.

[CR94] Pujadas E, Feinberg AP (2012). Regulated noise in the epigenetic landscape of development and disease. Cell.

[CR95] Ray J, Munn PR, Vihervaara A, Lewis JJ, Ozer A, Danko CG, Lis JT (2019). Chromatin conformation remains stable upon extensive transcriptional changes driven by heat shock. Proc Natl Acad Sci USA.

[CR96] Rubin AJ, Barajas BC, Furlan-Magaril M, Lopez-Pajares V, Mumbach MR, Howard I, Kim DS, Boxer LD, Cairns J, Spivakov M (2017). Lineage-specific dynamic and pre-established enhancer-promoter contacts cooperate in terminal differentiation. Nat Genet.

[CR97] Saldana-Meyer R, Rodriguez-Hernaez J, Escobar T, Nishana M, Jacome-Lopez K, Nora EP, Bruneau BG, Tsirigos A, Furlan-Magaril M, Skok J (2019). RNA interactions are essential for CTCF-mediated genome organization. Mol Cell.

[CR98] Schlesinger Y, Straussman R, Keshet I, Farkash S, Hecht M, Zimmerman J, Eden E, Yakhini Z, Ben-Shushan E, Reubinoff BE (2007). Polycomb-mediated methylation on Lys27 of histone H3 pre-marks genes for de novo methylation in cancer. Nat Genet.

[CR99] Schlesinger S, Kaffe B, Melcer S, Aguilera JD, Sivaraman DM, Kaplan T, Meshorer E (2017). A hyperdynamic H3.3 nucleosome marks promoter regions in pluripotent embryonic stem cells. Nucleic Acids Res.

[CR100] Shahzad U, Li C, Johnston M, Wang JJ, Sabha N, Varn FS, Riemenschneider A, Krumholtz S, Meda P, Smith CA (2020). CASCADES, a novel SOX2 super-enhancer associated long noncoding RNA, regulates cancer stem cell specification and differentiation in glioblastoma multiforme. bioRxiv.

[CR101] Shakya A, Callister C, Goren A, Yosef N, Garg N, Khoddami V, Nix D, Regev A, Tantin D (2015). Pluripotency transcription factor Oct4 mediates stepwise nucleosome demethylation and depletion. Mol Cell Biol.

[CR102] Shan J, Shen J, Liu L, Xia F, Xu C, Duan G, Xu Y, Ma Q, Yang Z, Zhang Q (2012). Nanog regulates self-renewal of cancer stem cells through the insulin-like growth factor pathway in human hepatocellular carcinoma. Hepatology.

[CR103] Shibata H, Komura S, Yamada Y, Sankoda N, Tanaka A, Ukai T, Kabata M, Sakurai S, Kuze B, Woltjen K (2018). In vivo reprogramming drives Kras-induced cancer development. Nat Commun.

[CR104] Sigova AA, Abraham BJ, Ji X, Molinie B, Hannett NM, Guo YE, Jangi M, Giallourakis CC, Sharp PA, Young RA (2015). Transcription factor trapping by RNA in gene regulatory elements. Science.

[CR105] Soeda A, Park M, Lee D, Mintz A, Androutsellis-Theotokis A, McKay RD, Engh J, Iwama T, Kunisada T, Kassam AB (2009). Hypoxia promotes expansion of the CD133-positive glioma stem cells through activation of HIF-1alpha. Oncogene.

[CR106] Spitz F, Furlong EE (2012). Transcription factors: from enhancer binding to developmental control. Nat Rev Genet.

[CR107] Stevens TJ, Lando D, Basu S, Atkinson LP, Cao Y, Lee SF, Leeb M, Wohlfahrt KJ, Boucher W, O’Shaughnessy-Kirwan A (2017). 3D structures of individual mammalian genomes studied by single-cell Hi-C. Nature.

[CR108] Szabo Q, Donjon A, Jerkovic I, Papadopoulos GL, Cheutin T, Bonev B, Nora EP, Bruneau BG, Bantignies F, Cavalli G (2020). Regulation of single-cell genome organization into TADs and chromatin nanodomains. Nat Genet.

[CR109] Therizols P, Illingworth RS, Courilleau C, Boyle S, Wood AJ, Bickmore WA (2014). Chromatin decondensation is sufficient to alter nuclear organization in embryonic stem cells. Science.

[CR110] Timp W, Bravo HC, McDonald OG, Goggins M, Umbricht C, Zeiger M, Feinberg AP, Irizarry RA (2014). Large hypomethylated blocks as a universal defining epigenetic alteration in human solid tumors. Genome Med.

[CR111] Towbin BD, Gonzalez-Aguilera C, Sack R, Gaidatzis D, Kalck V, Meister P, Askjaer P, Gasser SM (2012). Step-wise methylation of histone H3K9 positions heterochromatin at the nuclear periphery. Cell.

[CR112] Underwood JM, Becker KA, Stein GS, Nickerson JA (2017). The ultrastructural signature of human embryonic stem cells. J Cell Biochem.

[CR113] van Steensel B, Belmont AS (2017). Lamina-associated domains: links with chromosome architecture, heterochromatin, and gene repression. Cell.

[CR114] Vian L, Pekowska A, Rao SSP, Kieffer-Kwon KR, Jung S, Baranello L, Huang SC, El Khattabi L, Dose M, Pruett N (2018). The energetics and physiological impact of cohesin extrusion. Cell.

[CR115] Wang Y, Liu Y, Malek SN, Zheng P, Liu Y (2011). Targeting HIF1alpha eliminates cancer stem cells in hematological malignancies. Cell Stem Cell.

[CR116] Wang MC, Jiao M, Wu T, Jing L, Cui J, Guo H, Tian T, Ruan ZP, Wei YC, Jiang LL (2016). Polycomb complex protein BMI-1 promotes invasion and metastasis of pancreatic cancer stem cells by activating PI3K/AKT signaling, an ex vivo, in vitro, and in vivo study. Oncotarget.

[CR117] Wang H, Xu X, Nguyen CM, Liu Y, Gao Y, Lin X, Daley T, Kipniss NH, La Russa M, Qi LS (2018). CRISPR-mediated programmable 3D genome positioning and nuclear organization. Cell.

[CR118] Wang B, Kong L, Babu D, Choudhary R, Fam W, Tng JQ, Goh Y, Liu X, Song FF, Chia P (2020). Three-dimensional genome organization maps in normal haematopoietic stem cells and acute myeloid leukemia. bioRxiv.

[CR119] Wei X, Xiang Y, Abnousi A, Sun T, Lin X, Li W, Hu M, Diao Y (2020). HiCAR: a robust and sensitive multi-omic co-assay for simultaneous measurement of transcriptome, chromatin accessibility, and cis-regulatory chromatin contacts. bioRxiv.

[CR120] Weintraub AS, Li CH, Zamudio AV, Sigova AA, Hannett NM, Day DS, Abraham BJ, Cohen MA, Nabet B, Buckley DL (2017). YY1 Is a structural regulator of enhancer-promoter loops. Cell.

[CR121] Wen B, Wu H, Shinkai Y, Irizarry RA, Feinberg AP (2009). Large histone H3 lysine 9 dimethylated chromatin blocks distinguish differentiated from embryonic stem cells. Nat Genet.

[CR122] Whyte WA, Orlando DA, Hnisz D, Abraham BJ, Lin CY, Kagey MH, Rahl PB, Lee TI, Young RA (2013). Master transcription factors and mediator establish super-enhancers at key cell identity genes. Cell.

[CR123] Xiong X, Schober M, Tassone E, Khodadadi-Jamayran A, Sastre-Perona A, Zhou H, Tsirigos A, Shen S, Chang M, Melamed J (2018). KLF4, a gene regulating prostate stem cell homeostasis, is a barrier to malignant progression and predictor of good prognosis in prostate cancer. Cell Rep.

[CR124] Yochum GS, Sherrick CM, Macpartlin M, Goodman RH (2010). A beta-catenin/TCF-coordinated chromatin loop at MYC integrates 5’ and 3’ Wnt responsive enhancers. Proc Natl Acad Sci USA.

[CR125] You JS, Jones PA (2012). Cancer genetics and epigenetics: two sides of the same coin?. Cancer Cell.

[CR126] Zhang H, Li H, Xi HS, Li S (2012). HIF1alpha is required for survival maintenance of chronic myeloid leukemia stem cells. Blood.

[CR127] Zhu J, Adli M, Zou JY, Verstappen G, Coyne M, Zhang X, Durham T, Miri M, Deshpande V, De Jager PL (2013). Genome-wide chromatin state transitions associated with developmental and environmental cues. Cell.

